# Basic Research of *Plasmodium vivax* Biology Enabling Its Management as a Clinical and Public Health Problem

**DOI:** 10.3389/fcimb.2021.696598

**Published:** 2021-09-03

**Authors:** J. Kevin Baird

**Affiliations:** ^1^Eijkman-Oxford Clinical Research Unit, Eijkman Institute of Molecular Biology, Jakarta, Indonesia; ^2^Centre for Tropical Medicine and Global Health, Nuffield Department of Medicine, University of Oxford, Oxford, United Kingdom

**Keywords:** *Plasmodium vivax*, biology, pathophysiology, diagnosis, treatment, control

## Abstract

The emerging understanding of *Plasmodium vivax* as an infection seated in extravascular spaces of its human host carries fundamentally important implications for its management as a complex clinical and public health problem. This progress begins to reverse decades of neglected research borne of the false dogma of *P. vivax* as an intrinsically benign and inconsequential parasite. This Review provides real world context for the on-going laboratory explorations of the molecular and cellular events in the life of this parasite. Chemotherapies against the latent reservoir impose extraordinarily complex and difficult problems of science and medicine, but great strides in studies of the biology of hepatic *P. vivax* promise solutions. Fundamental assumptions regarding the interpretation of parasitaemia in epidemiology, clinical medicine, and public health are being revisited and reassessed in light of new studies of *P. vivax* cellular/molecular biology and pathogenesis. By examining these long overlooked complexities of *P. vivax* malaria, we open multiple new avenues to vaccination, chemoprevention, countermeasures against transmission, epidemiology, diagnosis, chemotherapy, and clinical management. This Review expresses how clarity of vision of biology and pathogenesis may rationally and radically transform the multiple means by which we may combat this insidiously harmful infection.

## Global *Plasmodium vivax*

Among the five protozoan species of plasmodia that naturally infect humans, none is more geographically widespread than *Plasmodium vivax* (Battle et al., 2019). It occurs across most of the perpetually warm tropics, but also where frigid winter seasons interrupt mosquito vector life, as on the Korean Peninsula. Only a century ago, *P. vivax* transmission regularly occurred across temperate Europe and North America, thriving particularly in the sub-tropical latitudes of those continents. Up to the present day, outbreaks of *P. vivax* occasionally occur in those zones receptive to it with seasonally abundant anopheline mosquitoes (Sunstrum et al., 2001; Andriopoulos et al., 2013).

The biology of *P. vivax* endows it physical characteristics and behaviours adapted to its broad geographic reach. These are both subtle and conspicuous, like sporogony in the mosquito proceeding at lower temperatures, and hepatic latency (Coatney et al., 1971). If the presence of anophelines occurs in relatively cool conditions and too briefly for a second cycle of transmission, some *P. vivax* strains pass on a futile primary attack and commit wholly to hepatic latency lasting to the next season of anopheline abundance (Garnham et al., 1975; Schute et al., 1976). Latency also occurs among tropical strains, but of short duration (White, 2011). Lysenko et al. (1977) rationally hypothesized selection for these relapse behaviours by the abundance or paucity of anophelines, i.e., forms of rapid latency vanished where and when mosquitoes appeared too briefly or not at all.

At the beginning of the current century, we believed much of Sub-Saharan Africa to be protected from endemic *P. vivax* transmission by the dominance of Duffy factor negativity among those residents. Red blood cells lacking Duffy factor seemed impenetrable to *P. vivax* merozoites, but the discovery of Duffy-negative Africans carrying *P. vivax* (Menard et al., 2010) challenged that view and sparked investigations of alternate erythrocyte invasion pathways (Kanjee et al., 2021). The increasing number of such infections being detected in Africa (Oboh et al., 2020; Dongho et al., 2021) raises the spectre of a parasite possibly evolving a workaround to Duffy negativity (Gunalan et al., 2018). Alternatively, we had not grasped a biology that underpins broad endemic transmission occurring without patent parasitaemia; Duffy-negative populations may have always harboured a silent and cryptic reservoir of infection and on-going endemic transmission. We could neither see it by conventional diagnostic means nor understand the inadequacy of those means to the biology of this parasite.

The global Northern clinical perspective focuses attention on the consequential acute attack, but in global Southern endemic zones patency in malaria is the exceptional state of infection (Sutanto et al., 2018; Hailemeskel et al., 2021). It is the silent reservoirs that dominate: sub-patent blood infections and latent hepatic infections. There may be great complexity in sub-patency, with extravascular compartments of infection beyond the peripheral blood window through which we have always viewed malaria. Likewise, the anatomic and temporal disposition of sexual gametocytes – observed and measured from anatomic sites inaccessible to probing anophelines – is another area of basic biological exploration of profound importance to rationally understanding and combatting the malarias. The primary technical challenge in eliminating malaria is not the task of diagnosis and treatment of people ill with malaria, but it is the management of people who are not acutely ill but nonetheless chronically infected, more subtly ill, and infectious to mosquitoes.

## States of Infection

**Figure 1:**
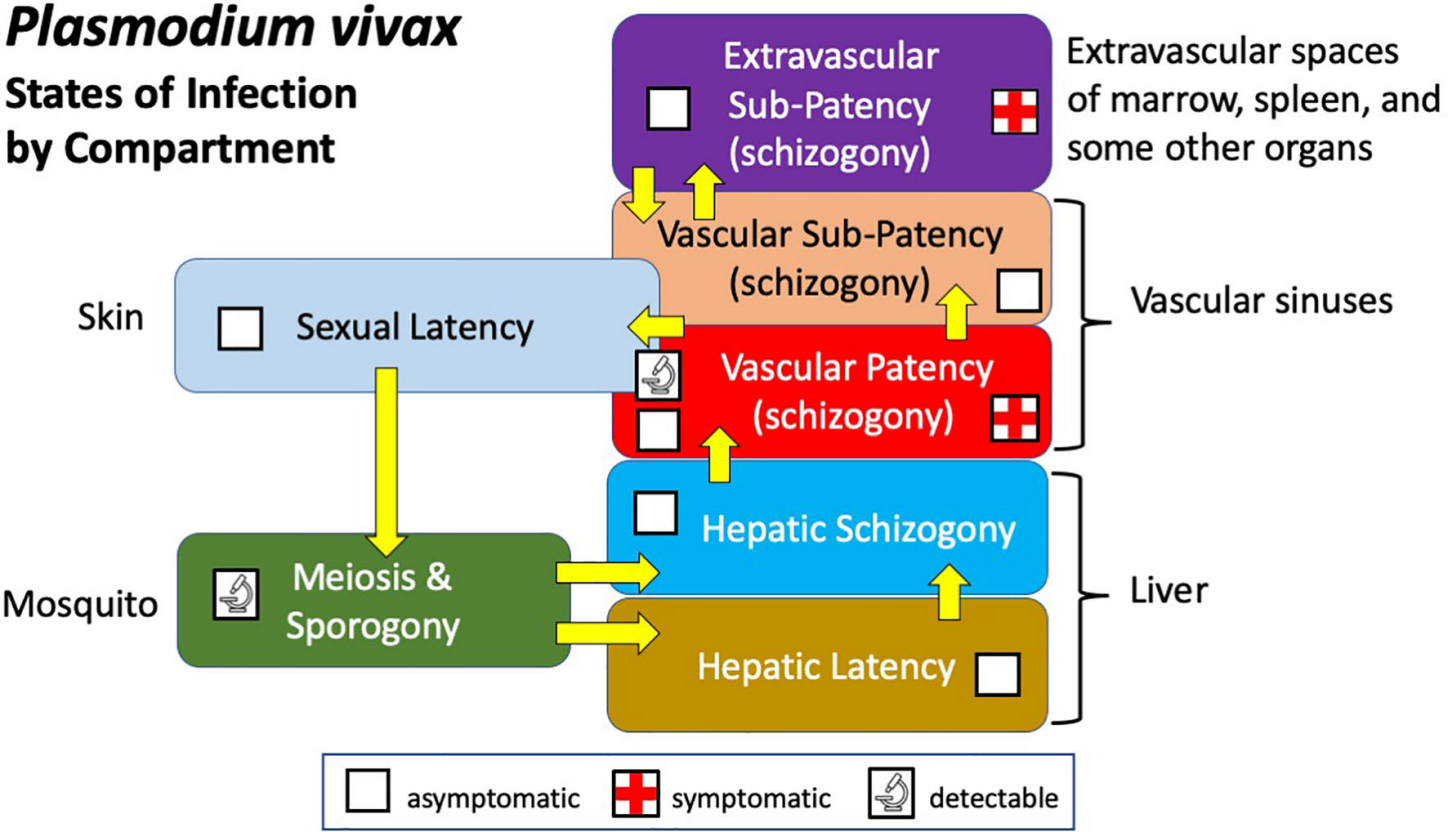
Diagram illustrating the states and compartments of infection by *P. vivax* in humans and mosquitoes.

Infection by *P. vivax* involves multiple compartments of varied biologics and clinical consequences (**Figure 1**). Sporogony follows sexual union and meiosis in the mosquito gut, yielding infectious sporozoites leading directly to hepatic schizogony (by tachysporozoites) or latent hypnozoites (by bradysporozoites) (Lysenko et al., 1977). Hepatic infection, which is wholly silent and cannot be detected, leads to the vascular patency of blood schizogony which may be asymptomatic or provoke an acute attack of clinical malaria. That malaria is typically readily diagnosed (i.e., patent) by microscopy, antigen capture, or PCR techniques applied to peripheral blood. Semi-immune hosts suppress parasitaemia to below conventionally detectable levels (i.e., sub-patent) and acute illness rarely occurs. Recent work suggests that much of the biomass of asexual trophozoites and schizonts of *P. vivax* occur in the extravascular spaces of marrow, spleen, and liver (Siquiera et al., 2012; Malleret et al., 2015; Obaldia et al., 2018; Fernandez-Becerra et al., 2020; Kho et al., 2021). Though not yet demonstrated, it is plausible that this relatively inaccessible compartment may both contribute to acute illness (Lacerda et al., 2012) and sometimes be clinically silent (Siquiera et al., 2012; Kho et al., 2021). In either case, extravascular sub-patency almost certainly places infected red blood cells back into the vascular sinuses and peripheral circulation, be those patent or sub-patent.

Vascular infection includes sexual gametocytes and placement of them within reach of feeding anophelines, invariably no more than two or three millimetres below the epidermis. This state of infection may be referred to as sexual latency in the sense that gametocytes, like hypnozoites, are virtually but not entirely metabolically inactive and invariably clinically silent (Baker, 2010; Gural et al., 2018). Although the evidence is as yet scanty (Nixon, 2016), long-term presence of these nearly dormant gametocytes lodged within the uppermost reaches of the dermal capillaries is biologically plausible and in an evolutionary sense, tremendously advantageous. Infection of naturally feeding mosquitoes occurs in patients without patent gametocytaemia (Bousema and Drakeley, 2011), and parallel feeds of mosquitoes on skin or membranes (containing venous blood) consistently show heavier and more frequent infections from skin (Sattabongkot et al., 2003; Bousema et al., 2012; Meibalan et al., 2019). Enduring sexual latency of skin microvasculature may be a vitally important compartment of infection. The favoured selection of forms capable of this behaviour making them available to feeding anophelines over prolonged periods may be intuitively obvious.

## Harmful Latent Reservoir

We cannot diagnose latency in *P. vivax* and thus cannot know its prevalence in endemic zones. It is a condition that may be recognized as having been present only upon patency. Nonetheless, the biology of this infection, where Lysenko’s tachysporozoites and bradysporozoites (Lysenko et al., 1977) seed the liver, rationally informs an expectation of a prevalence of latency in excess of that measured by mass blood screening by microscopy, antigen capture, or PCR methods. Longitudinal cohort studies of children randomised to treatment of latency or placebo in Papua New Guinea (Robinson et al., 2015) suggested that approximately 80% of *P. vivax* attacks derived not from recent mosquito bites, but activation of hepatic latency. Similar cohort studies in western Thailand estimated that proportion at over 90%, and estimated that each person infected by *P. vivax* carried, on average, five hypnozoites in their livers (Adekunle et al., 2015). A review and meta-analysis of 261 studies of over four thousand patients from seven countries in Africa and Asia estimated a minimum of 79% of patent infections from relapses (Commons et al., 2020). In mathematical models of *P. vivax* that include this hypnozoite reservoir, transmission quickly collapses when it is successfully assaulted (Roy et al., 2013; White et al., 2014). For reasons explained below, doing so presents formidable challenges, and in most endemic zones today it does not effectively occur. The hypnozoite reservoir streams new clinical attacks, sub-patent infections, and onward transmission into the communities where it stands.

The harm done by the hypnozoite reservoir may be subtle but nonetheless appears to be substantial. Studies carried out near Timika in eastern Indonesia shed light on this harm. Over a period of ten years, Dini and colleagues (Dini et al., 2020) followed cohorts of tens of thousands of patients infected by *P. vivax* or *P. falciparum*. While risk of hospitalisation and mortality within two weeks of the index infection was higher for *P. falciparum*, as months and years passed, those diagnosed with *P. vivax* endured more repeated attacks, hospitalizations, and higher risk of all-cause mortality compared to the *P. falciparum*-infected cohort. Endemic malaria of repeated clinical attacks imposes poorly understood dangers to patients.

The global Northern perspective deceives by exclusive focus on the event of the acute attack. Good health is interrupted by a patent infection and febrile illness – prompt diagnosis and effective treatment restores health, and we are done. The global Southern reality is more complex than that. Chronic exposure to malaria - patent and sub-patent arising from mosquitoes or latency - carries health consequences beyond the paroxysms of acute malaria (Chen et al., 2016; Lee and Corban, 2018). Malaria is not just a clinical event of days, but a continuum of events across years of cumulative and more subtle harm. Fragile states of health and healthcare delivery make that continuum all the more consequential. In this global Southern perspective, we may grasp the essential importance of successfully assaulting the latent reservoir of *P. vivax*.

## Latent Reservoir Donjon

A donjon is the innermost keep of a fortified castle, the most difficult and last thing to take from its defenders. Untaken, the assault fails and the defenders recover. The latent reservoir of *P. vivax* may be thus reckoned. What follows below are the complex and formidable obstacles that must be overcome in delivering a successful assault on that hard defence. Each is unique in character, but most of them are linked to inherent pitfalls in the 8-aminoquinolines, the only class of compounds we have that kill latent hypnozoites. Our only offensive weapon against hypnozoites is deeply flawed for use where it is most often needed – the often impoverished rural settings where endemic malaria endures.

### Stealth

Invisibility is perhaps the greatest defence of the latent reservoir of *P. vivax* against our assaults upon it. In any given endemic area we cannot know who or how many people carry hypnozoites. Typically, an infected person will harbour just a few of them, each one cocooned within a functioning hepatocyte and wholly silent. Those that awaken and provoke renewed attacks do so alongside new mosquito-borne infections, and we cannot distinguish them by genetic analyses. Deceptively low parasitaemias in acute vivax malaria conceals a larger and more harmful biomass, and chronic infection brings more subtle harm. Latency is hidden and the patency it delivers is disguised and deceptive. Mustering the will to assault such a difficult target requires first recognizing it as a threat that merits the effort and risk thus engaged. In most endemic zones, the great stealth of latency and its harm very substantially impedes the ability and will to attack it.

### Human G6PD Deficiency

The haemolytic toxicity of the 8-aminoquinolines in patients having the common inherited enzymopathy glucose-6-phosphate dehydrogenase (G6PD) deficiency severely constrains the clinical and public health management of the *P. vivax* problem (Howes et al., 2013). G6PD deficiency is not a single polymorphism of the enzyme, but many dozens of them (Luzzatto et al., 2020) of variable sensitivity to 8-aminoquinolines (Baird, 2015). It is likely that we have misconstrued the variants of G6PD deficiency in a dichotomy of relatively mild and inconsequential versus severe and life-threatening. All of the variants are sensitive to 8-aminoquinolines, with life threatening drug-induced severe acute haemolytic anaemia occurring even in patients with so-called mild variants (Pamba et al., 2012; Monteiro et al., 2016). The clinical complexity of the event in part explains the danger of oversimplified rules of thumb regarding this serious problem (Chu and Freedman, 2019; Bancone and Chu, 2021).

The clinical dilemma created by G6PD deficiency and 8-aminoquinolines is clear. Providers standing before patients diagnosed with *P. vivax* and having unknown G6PD status must make a choice between these two stark options: 1) prescribe anti-relapse therapy and risk acute haemolytic anaemia; or 2) withhold therapy and risk further clinical attacks and opportunities for harm and onward transmission. How national malaria control programmes manage this dilemma varies widely (Recht et al., 2018). The global Northern perspective of ready access to G6PD screening, and to care in the event of haemolytic crisis largely explains the neglect of the haemolytic toxicity problem through three cycles of 8-aminoquinoline discovery efforts through the past century (Baird, 2019). We don’t know if non-haemolytic 8-aminoquinolines exist because, remarkably, we have never surveyed those compounds for this characteristic. Resolving the therapeutic dilemma today hinges on G6PD screening before administering the haemolytic 8-aminoquinolines available to us (Anderle et al., 2018). Success in doing so is the first offensive breach on the hypnozoite reservoir.

### 8-Aminoquinoline Chemotherapeutics

The 8-aminoquinolines impose other problems that serve to protect the latent reservoir of *P. vivax*. Chief among those may be the lack of understanding of how these compounds actually kill latent hypnozoites in patients who may safely receive them. The discovery of 8-aminoquinolines as hypnozoitocidal in the early 1920s was wholly serendipitous, with activity against “delayed attacks” (latent forms were suspected but unknown) of *P. vivax* coming unexpectedly with the first rationally synthesized drug, plasmochin, designed and optimized to treat acute attacks (Baird, 2019). Dangerous haemolytic toxicity in some patients forced providers to augment diminished doses of plasmochin with quinine, unwittingly inventing what we call radical cure, i.e., a hypnozoitocide combined with a blood schizontocide to cure patent and latent forms of the infection (Dini et al., 2020). Sinton and colleagues (Sinton and Bird, 1928; Sinton et al., 1930) invented and optimized radical cure of *P. vivax* 90 years ago, about 50 years before the existence of hepatic hypnozoites was confirmed (Krotoski, 1985).

The impact of blood schizontocides on 8-aminoquinoline hypnozoitocidal activity was recognized in that early era of discovery (Sinton et al., 1930) and confirmed 25 years later (Alving et al., 1955). Like other blood schizontocides, quinine alone has no impact on *P. vivax* latency, but it dramatically improved the activity of plasmochin or primaquine against hypnozoites (Baird, 2019). Chloroquine, equally inactive against hypnozoites, also did so (Alving et al., 1955). Potentiation of the modern 8-aminoquinoline called tafenoquine was observed in the model of *Plasmodium cynomolgi* in rhesus macaques (Dow et al., 2011). The clinical development of tafenoquine involved no other partner blood schizontocides but chloroquine (Lacerda et al., 2019), but when the US Food and Drug Administration (FDA) approved tafenoquine for radical cure of *P. vivax* in 2018 it recommended any “appropriate” partner blood schizontocidal therapy. When tafenoquine was combined with dihydroartemisinin-piperaquine in a clinical trial of *P. vivax* radical cure in Indonesia – where chloroquine cannot be applied due to parasite resistance to it (Price et al., 2014) – there was almost no therapeutic efficacy (Centers for Disease Control and Prevention (CDC), 2020). Efficacy in radical cure may not accommodate flexibility in applying partner blood schizontocides with 8-aminoquinolines. Assault on the hypnozoite reservoir will require tools that actually work; that is, combinations of blood schizontocides and hypnozoitocides of optimized and validated safety and efficacy.

Another fundamental problem of 8-aminoquinoline chemotherapeutics is the uncertainty of effective dosing. In striving to mitigate risk of harm to G6PD-unknown patients, we have consistently applied doses at the margin of good efficacy. This has been true of primaquine (Baird and Hoffman, 2004), and remains true of tafenoquine (Hanboonkunupakarn and White, 2020). Intrinsically variable susceptibility to 8-aminoquinolines among strains of *P. vivax* has long been recognized (Collins and Jeffery, 1996) and authoritative guidance to apply universally more effective higher dosing regimens (Hill et al., 2006) are not broadly accepted and implemented (Recht et al., 2018). Hesitancy, uncertainty, and confusing inconsistency characterizes 8-aminoquinoline treatment policies and practices. This weakness serves to protect the latent reservoir.

### Human Metabolism of 8-Aminoquinolines

The problematic complexity of radical cure applying 8-aminoquinolines is greatly compounded by the necessity of metabolic activation of those compounds by the patient. The therapeutic activity of these drugs seem to require hydroxylation of the 5-position of the aminoquinoline ring (Marcsisin et al., 2016). In the instance of primaquine, that involves human cytochrome P450 2D6 (CYP2D6) (Bennett et al., 2013), a naturally polymorphic isozyme of activities ranging from null- to ultra-metabolizer phenotypes of significant clinical consequences for very many therapeutic agents (Nofziger et al., 2020). Patients having impaired metabolic activity of CYP2D6 are at much higher risk of therapeutic failure of primaquine against relapse (Baird et al., 2018). The extraordinarily high frequency of the impaired *10 allele in Asia may compromise primaquine efficacy (Spring et al., 2020). Tafenoquine does not appear to be CYP2D6-dependent (St Jean et al., 2016), but it may nonetheless require activation by other means; its very poor efficacy when administered with dihydroartemisinin (Centers for Disease Control and Prevention (CDC), 2020) suggests interference with that activation. The complexities of human metabolism of 8-aminoquinolines compound the difficulty of assaulting the hypnozoite reservoir.

### 8-Aminoquinoline Ineligibles

The people who cannot safely receive or benefit from 8-aminoquinoline therapies constitute a safe haven for latent *P. vivax* (Baird et al., 2018). Foremost among them in numbers may be the G6PD-deficient at risk of harm by therapy, and the CYP2D6-impaired at risk of poor efficacy. Those are permanent conditions in many hundreds of millions of people living at risk of infection, whereas those ineligible by pregnancy or infancy are counted in many tens of millions and transiently ineligible. Some authorities include lactating women as ineligible, probably unnecessarily (Gilder et al., 2018). There is no validated approach to managing risk of relapse in these patients, many of them being especially vulnerable to harm caused those acute attacks of vivax malaria (McGready et al., 2012; Rijken et al., 2012; Kenangalem et al., 2016). A far better understanding of hypnozoite basic biology could perhaps yield a means of activating them in patients like these placed under reasonably brief protective suppressive chemoprophylaxis (rather than impractically for a year).

## Insidious *Plasmodium vivax* Malaria

### Acutely Ill Patients

In 1949, S.F. Kitchen writing in Mark Boyd’s classical text of modern malariology, explicitly described *P. vivax* as intrinsically benign (Kitchen, 1949). Remarkably, he did so while also describing severe anaemia, hyperpyrexia, seizures, splenomegaly, severe dehydration, intractable vomiting, and astasis as common consequences of acute *P. vivax*. Kitchen’s patient subjects were undergoing induced vivax malaria as therapy for otherwise invariably fatal neurosyphilis. Quinine therapy was withheld or minimized in order to allow maximum severity of the paroxysms in order to improve the relatively poor efficacy of those attacks against neurosyphilis (only about 30% in the best clinics). Simply put, the patient’s life depended on the severity of malaria experienced. Although Kitchen did not mention any of his patients not surviving that therapy (or the neurosyphilis prompting it), other practitioners reported fatality rates for induced vivax malaria that typically ranged from 5-15% (Baird, 2013). According to Nicol (1927), who treated many hundreds of such patients in the UK, it was acute vivax malaria killing patients under treatment rather than complications due to neurosyphilis or other comorbidities. Autopsy findings supported that view (Fong, 1937).

**Figure 2:**
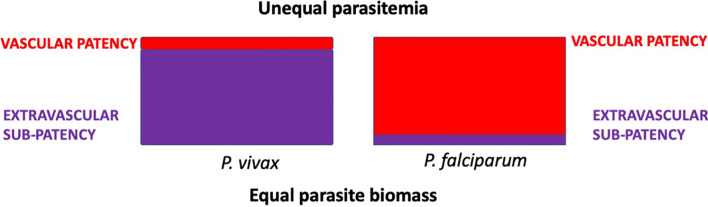
Hypothetical distribution of equal parasite biomass (areas of the rectangles) with infection by *P. vivax* or *P. falciparum* in acutely ill patients. Tropisms of *P. vivax* disfavour the intravascular compartment, whereas those of *P. falciparum* place it predominantly within those sinuses. Estimates of parasite biomass based on counts of parasites observed in smears of peripheral blood would be disproportionately underrepresented in *P. vivax* malaria.

Another observation from Nicol and his colleagues is relevant. They also treated a minority of many dozens of patients with induced *Plasmodium falciparum* malaria, among whom death during therapy occurred in about 4%, whereas among his patients treated with the Madagascar strain of *P. vivax*, it was 14% (James et al., 1932). They referred to these species in this context as causing “so-called malignant” and “so-called benign” malarias. A possible explanation for this seemingly paradoxical observation is relatively simple and evidence for it is mounting. The bulk of harmful parasite biomass may not occur in the readily observable peripheral blood within vascular sinuses but in the relatively inaccessible and unobserved extravascular spaces of some deep organs like bone marrow and spleen. In reading the old malaria neurosyphilis therapy literature, the clinician’s struggle to keep the patient alive through repeated bouts of deliberately severe malaria paroxysms is conspicuous and intuitively obvious. Those attending assiduously monitored microscopic parasitemia counts in peripheral blood smears in nearly real time - intelligence that guided the suppression of those with measured sub-curative doses of quinine when perceived as dangerously high. The inferior malaria therapy survival rates among patients with induced *P. vivax* relative to *P. falciparum* may have been a consequence of unseen and unmanaged *P. vivax* biomass. The schematic diagram of **Figure 2** illustrates this hypothesized explanation.

Sub-patency in acute vivax malaria is not simply a matter of low-grade parasitemias, but also of harmful parasite biomass effectively hidden from view beyond the vascular sinuses. As Nicol’s paradox of malignant and benign identity attests, and modern studies tend to affirm, severe and complicated acute *P. vivax* malaria is typically attended by very modest levels of parasitaemia (Silva-Filho et al., 2020). This species is not intrinsically benign, but inherently obscure and insidiously harmful.

### Chronically Infected Without Acute Illness

**Figure 3:**
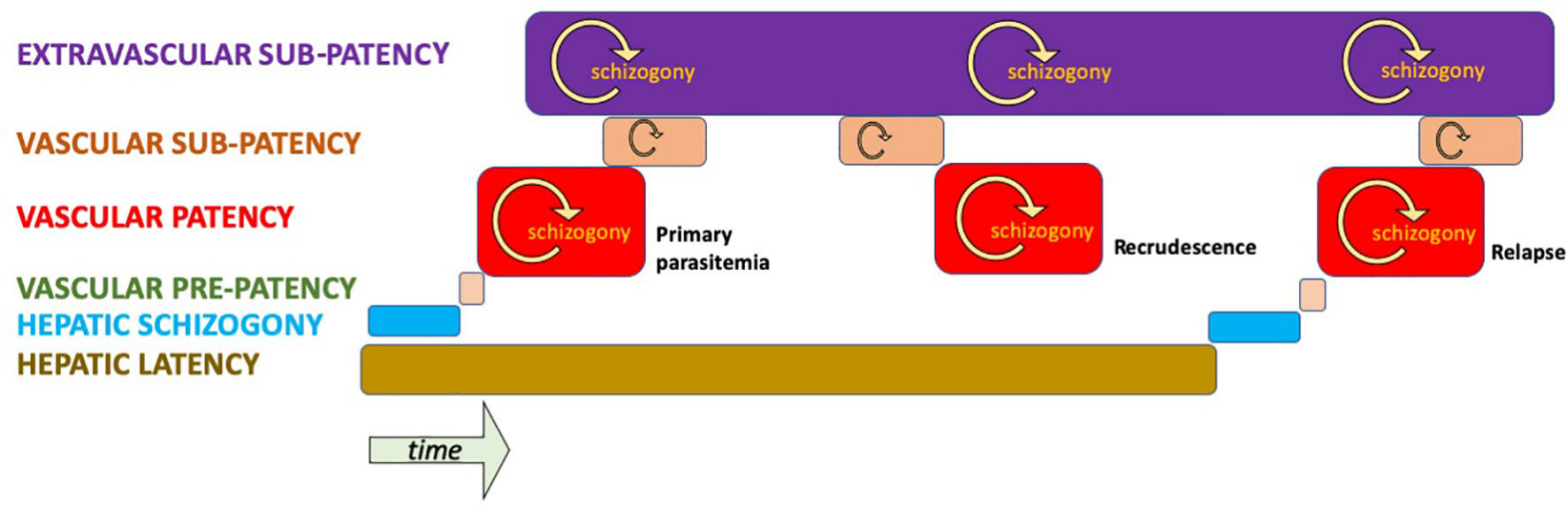
Hypothetical infection by *P. vivax* of a semi-immune host illustrating stages and compartments of infection. Only vascular patency affords an opportunity for diagnosis and treatment by active case detection in an asymptomatic carrier.

As has been explained, good evidence points to illness and patency as an exceptional state of infection among people living with endemic transmission. That may occur as a result of innate (e.g., Duffy negativity) or acquired immunity. **Figure 3** illustrates a hypothetical infection by *P. vivax* in a semi-immune host resulting in a primary parasitaemia and a single relapse. At the beginning of the infection (left of diagram), hepatic schizogony commences, as does the condition of latency by co-inoculation of tachy- and brady-sporozoites. A brief period of pre-patency in blood later occurs, leading to patent parasitaemia that is or becomes suppressed and sub-patent. Extravascular sequestration occurs in connection with the primary parasitaemia and may later release newly infected red blood cells back into the vascular sinuses, leading to a patent or sub-patent recrudescence. The last hypnozoite in the liver activates, ending latency and commencing another round of schizogony in infected red blood cells; be those patent, sub-patent or sequestered beyond the vascular sinuses. In this hypothetical semi-immune host, the condition of vascular or extravascular sub-patency along with sexual latency continues indefinitely without acute illness or therapy for the infection.

**Figure 4:**
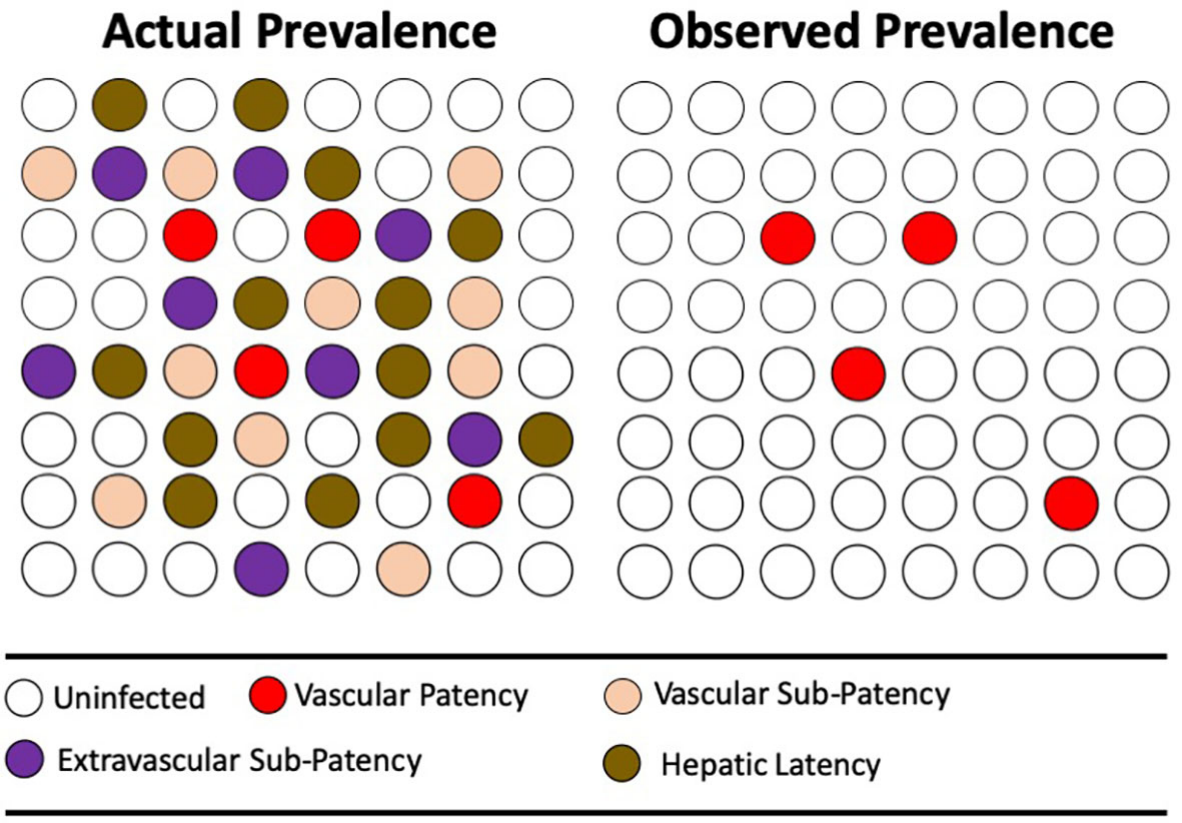
The diagrams represent the same population of hypothetical individuals being either uninfected (no color) or infected (color). The colors represent varied states of infection, where mass screening for microscopic or antigen-capture patency of infection (red) reveals the minority thus infected. More sensitive PCR detection reveals otherwise sub-patent infection of blood, but not extravascular sequestration of active parasites or the inactive parasites of hepatic latency.

These hypothesized distributions of *P. vivax* in varied compartments of its human host bears directly on the means by which we strive to measure burdens of infection. That is, up to the present day, by examination of the peripheral blood for evidence of the parasite; be it microscopically, by antigen capture, or PCR. What fraction of infected people are missed by these techniques cannot be known with certainty, and that fraction would certainly vary among distinct transmission settings. Nonetheless, evidence like that already discussed (dominant states of infections in endemic areas) mounts that most infections are indeed missed by mass blood screening by any of those techniques. **Figure 4** illustrates the problem hypothetically.

## Exploration of *Plasmodium vivax* Biology and Pathogenesis

This review has summarised the problems imposed by the biological and physiological character of *P. vivax* infection on the ways and means by which we strive to measure, avert, or mitigate the harm done. The subsections that follow describe how leveraging explorations of biology and pathogenesis may guide us to approaches of greater safety, convenience, and effectiveness underpinned by ever-greater scientific certainty and essential confidence in combatting that harm.

### Basic Biology Informing Improved Diagnostics

Accepting *P. vivax* as improbably detected by conventional diagnostic approaches among asymptomatic carriers using peripheral blood sampling opens multiple avenues of promising investigation. An epidemiology of vivax malaria based on classically diagnosed acute attacks is a Northern bias of clinical medicine and public health. Although the acutely ill are indeed reliably diagnosed by peripheral blood examination, the reality in endemic settings is the exceptional character of that patency with regard to the parasitism of human communities. Understanding endemic *P. vivax* epidemiology will require far greater diagnostic reach. That need properly drives the search for other indicators of infection, be they molecules of parasite or host origin.

Diagnostics underpin the clinical management of infectious diseases. Detecting and quantifying molecules derived from *P. vivax* may better guide determining the extent of parasitism of clinically ill patients. Plasmodial lactate dehydrogenase (pLDH) has been studied as a biomarker of total parasite biomass in patients infected by *P. vivax* (Barber et al., 2015). The ratio of that molecule to peripheral parasitaemia was 6-fold higher in patients with severe illness compared to relatively mild *P. vivax* malaria, whereas only slight differences occurred in that ratio between such patients with *P. falciparum* malaria (Barber et al., 2015; Silva-Filho et al., 2020). Reliance on parasite counts in peripheral blood smears may be dangerously deceptive with acute *P. vivax* malaria. Validating markers like pLDH may better guide the clinical management of patients with this infection.

Diagnostics also underpin the epidemiology of infectious diseases. Investigations of the serological epidemiology of malaria have been undertaken (Cook et al., 2010; Folegatti et al., 2017; Longley et al., 2020; Wu et al., 2020), but the presence of parasites in peripheral blood (by microscopy or antigen-capture) remains the standard of malaria epidemiology globally (World Health Organization, 2020). Mass surveys of populations involving specific validated antibodies of short-lived duration may reveal nearer-to-true prevalence of this infection. It remains possible, for one example, that sustained endemic transmission of *P. vivax* occurs in much of Sub-Saharan Africa where vascular patency – and therefore prevalence by conventional diagnostics – appears virtually absent. Serological surveys for *P. vivax* in areas of Sub-Saharan Africa where the infection is rarely found by conventional means ranged between about 10-60% positivity (Culleton et al., 2009; Poirier et al., 2016; Niang et al., 2017). Although some blood surveys employing PCR diagnostics also detected prevalent *P. vivax* (1%-15%) in the same region (Motshoge et al., 2016; Haiyambo et al., 2019; Oboh et al., 2020; Podgorski et al., 2020; Dongho et al., 2021), serological surveys may ultimately be required to detect and measure the extent of endemic *P. vivax* transmission.

### Basic Biology Informing Improved Interventions

Successful treatment of *P. vivax* requires combining an 8-aminoquinoline against latency with blood schizontocidal agents against patency. As already explained, the complexity of this task involves managing G6PD vulnerability, patient metabolism of 8-aminoquinolines, drug-drug interactions impacting safety and efficacy, and naturally variable parasite sensitivity to those drugs. In combining blood schizontocidal agents that optimize 8-aminoquinoline activity we may finally see validated radical cures of superior efficacy and maximal safety in most patients infected by any strain of *P. vivax* (Baird, 2019).

The tool needed to explore those combinations and strains is *ex vivo* hepatic systems accommodating not only immediate and delayed hepatic development of parasites, but also including the crucial metabolism/interactions of those drugs as it is likely to occur in patients. The many dozens of possible combinations, multiplied by many more dosing strategies, are too numerous to conceivably approach with clinical trials or even the *P. cynomolgi* in rhesus macaque model. Reasonably high throughput screening in the laboratory requires sophisticated biological systems of human (or macaque) hepatocyte culture coupled to ready access to viable sporozoites (Gural et al., 2018; Subramani et al., 2020; Voorberg van der Wel et al., 2020). That access would be vastly improved by laboratory systems supporting *in vitro* development of *P. vivax* blood schizogony and gametocytogenesis (Gunalan et al., 2020).

Those *ex vivo* systems, along with mice grafted with human liver (Schafer et al., 2020), offer a great deal more than screening drugs and exploring their mechanisms of action. Fundamentally important questions of *P. vivax* biology may also be addressed. Chief among those may be the phenomenon of hypnozoite formation, dormancy, and activation. Some of this *ex vivo* and rodent work, for example, corroborates Lysenko’s (Lysenko et al., 1977) hypothesis of polymorphic sporozoites, i.e., immediate versus delayed hepatic schizogony of what may be genetically determined periodicity (Mikolajczak et al., 2015). Those genetics await definitive phenotyping empowered by these new tools, along with increasingly available *P. vivax* whole genome and RNA transcriptome capacities (Bright et al., 2014; Brashear et al., 2020; Buyon et al., 2020; Ford et al., 2020; Bourgard et al., 2021). Better understanding of the molecular mechanics of hypnozoite biology may rationally inform interventions against them that we may scarcely imagine today.

Recent breakthroughs in the biology of *P. vivax* erythrocyte invasion mechanics deepen our understanding of pathogenesis and epidemiology, along with identifying specific potential targets of chemotherapy or vaccination (Rawlinson et al., 2019; Chan et al., 2020). Likewise, new work in the immunology of infection by *P. vivax* inform strategies for improved clinical management, new epidemiological tools in the form of markers of infections, and rational vaccine development (Antonelli et al., 2019). This important work includes the sexual forms of *P. vivax* and their singular biology and immunology (de Jong et al., 2019).

**Figure 5:**
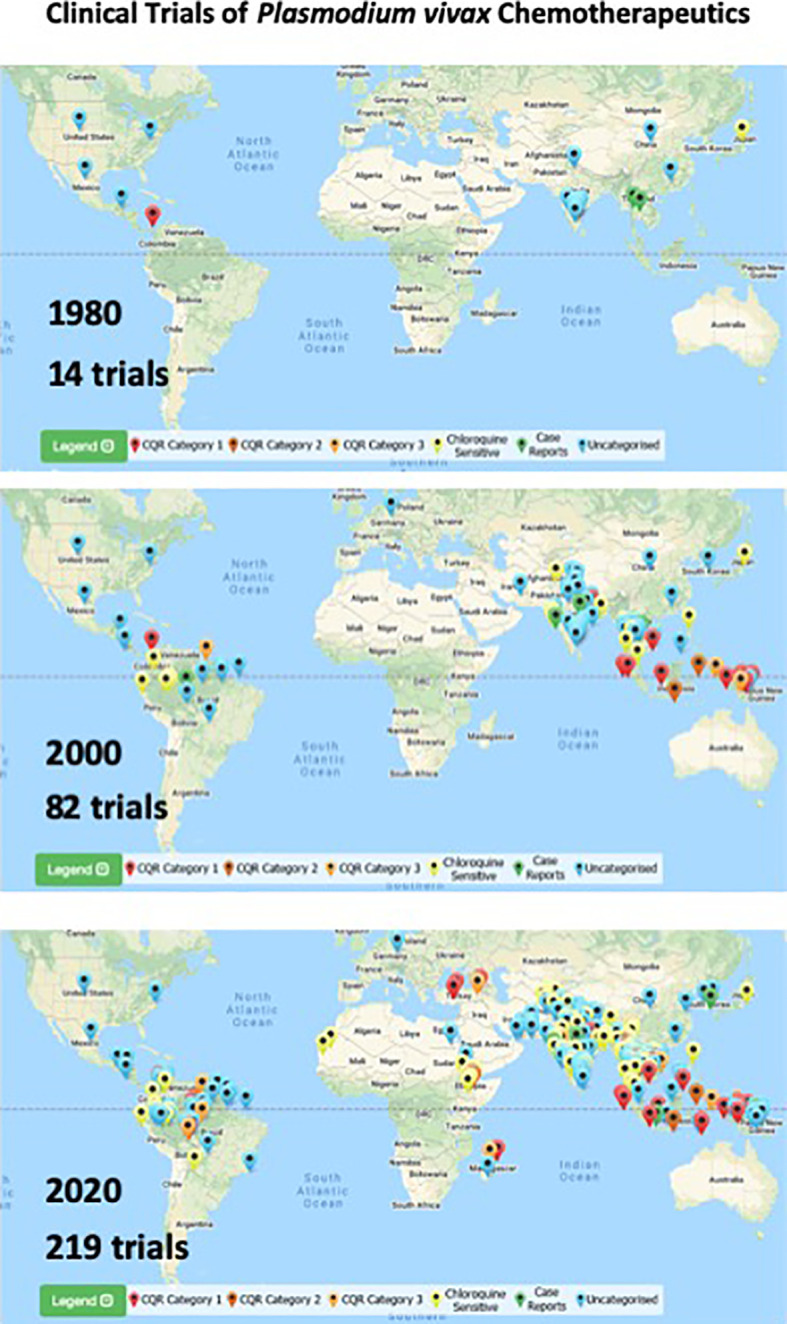
Maps illustrating location and cumulative numbers of clinical trials of chemotherapeutics involving *P. vivax* infected-patients up to 1980, 2000, and 2020 (top to bottom). Pin colors correspond to categories of responses to chloroquine therapy as indicated in the key at bottom of each map. Images and numbers taken from Vivax Surveyor on 8 April 2021 (http://www.wwarn.org/vivax/surveyor/#0).

In the field, clinic, and laboratory, research on *P. vivax* has greatly accelerated since 30 years ago when very little work was being done on this infection. Each year through the 1980s and early 1990s no more than 3 clinical trials involving *P. vivax* were reported, and during many of those years it was none at all (Commons et al., 2017). After the year 2000, that number approached or exceeded a dozen trials each year. **Figure 5** presents data from Vivax Surveyor (http://www.wwarn.org/vivax/surveyor/#0) illustrating this trend. Likewise, a PubMed search of “*Plasmodium vivax*” shows similar trends: through the 1980s and 1990s; around 100 citations appeared each year, but after 2000 a rapid rise to around 500 citations/year appears. These trends are intermingled, each spurring the other with new questions and demands for further work and progress.

## Summary

The long neglect of research on *P. vivax* as a clinical and public health problem now offers rich opportunities for penetrating explorations of basic biology and pathogenesis of direct relevance to preventing and mitigating the insidious harm of this infection. The global epidemiology of this infection may be very poorly understood, or even misunderstood by superficial diagnostic technologies inadequate to the biology of *P. vivax* – prevalence of parasitaemia may not reliably approximate prevalence of infection. Clinical management of acutely ill patients may be similarly misinformed by the same diagnostics – low-grade parasitaemia does not equate to low-grade infection. The complexity of the chemotherapeutic problem of radical cure has been underestimated and remains fraught with uncertainty, hazard, and hesitancy – available regimens are often not validated, inadequate, and sometimes dangerous. All of these serious gaps in understanding may be addressed by the basic research like that summarized in this issue of *Frontiers*. Acknowledging the stealth and complexity of *P. vivax* malaria may propel that hard work to very substantial progress against this important infection.

## Author Contributions

The author confirms being the sole contributor of this work and has approved it for publication.

## Funding

The author is supported by the Wellcome Trust Africa Asia Programme Vietnam.

## Conflict of Interest

The author declares that the research was conducted in the absence of any commercial or financial relationships that could be construed as a potential conflict of interest.

## Publisher’s Note

All claims expressed in this article are solely those of the authors and do not necessarily represent those of their affiliated organizations, or those of the publisher, the editors and the reviewers. Any product that may be evaluated in this article, or claim that may be made by its manufacturer, is not guaranteed or endorsed by the publisher.
